# Toxicity Profiles *In Vivo* in Mice and Antitumour Activity in Tumour-Bearing Mice of Di- and Triorganotin Compounds

**DOI:** 10.1155/MBD.1998.83

**Published:** 1998

**Authors:** M. Gielen, R. Willem, H. Dalil, D. de Vos, C. M. Kuiper, G. J. Peters

**Affiliations:** 1 Free University of Brussels VUB Faculty of Applied Sciences, Room 8G512 Pleinlaan 2 Brussels B-1050 Belgium; 2 Free University of Brussels VUB High Resolution NMR Centre Pleinlaan 2 Brussels B-1050 Belgium; 3 Medical Department Pharmachemie B. V. Haarlem The Netherlands; 4 Department of Oncology Laboratory of Biochemical Pharmacology University Hospital VU de Boelelaan 1117 Amsterdam NL- 1081 HV The Netherlands

## Abstract

The *in vivo* toxicity profiles in mice and the antitumour activity in tumour bearing mice were screened for four di-n-butyltin and five triorganotin carboxylates, di-n-butyltin diterebate (**5**), bis(phenylacetate) (**6**), bis(deoxycholate) (**7**), bis(lithocholate) (**8**), tri-n-butyltin terebate (**9**), cinnamate (**10**), and triphenyltin terebate (**11**).

At their maximum tolerated dosis (MTD), no antitumour effect (T/C ~1) was observed for the compounds **5**, **7**, **9**, **10** and **11**. The compounds **6** (T/C = 0.51) and **8** (T/C = 0.42) showed clear antitumour activity after single dose administration and might therefore be of interest for further antitumour activity studies.


### TOXICITY PROFILES *IN VIVO* IN MICE AND ANTITUMOUR ACTIVITY IN TUMOUR-BEARING MICE OF DI- AND TRIORGANOTIN COMPOUNDS

M. Gielen*[1a], R. Willem[1a, 1b], H. Dalil[1a], D. de Vos[2], C. M. Kuiper[3], and G. J. Peters[3]

[1] Free University of Brussels VUB,
a) Faculty of Applied Sciences, Room 8G512, b) High Resolution NMR Centre
Pleinlaan 2, B-1050 Brussels, Belgium
[2] Medical Department, Pharmachemie B. V., Haarlem, The Netherlands
[3] Department of Oncology, Laboratory of Biochemical Pharmacology, University Hospital VU,
de Boelelaan 1117, NL - 1081 HV Amsterdam, The Netherlands

**Abstract.**
The *in vivo* toxicity profiles in mice and the antitumour activity in tumour bearing mice were screened for four di-n-butyltin and five triorganotin carboxylates, di-n-butyltin diterebate (**5**), bis(phenylacetate) (**6**), bis(deoxycholate) (**7**), bis(lithocholate) (**8**), tri-n-butyltin terebate (**9**), cinnamate (**10**), and triphenyltin terebate (**11**).
At their maximum tolerated dosis (MTD), no antitumour effect (T/C ~1) was observed for the compounds **5**, **7**, **9**, **10** and **11**. The compounds **6** (T/C = 0.51) and **8** (T/C = 0.42) showed clear antitumour activity after single dose administration and might therefore be of interest for further antitumour activity studies.

### Introduction

Organotin carboxylates often exhibit significant *in vitro* antitumour activities (Bouâlam, 1992)(Bouâlam, 1993)(Gielen, 1996a)(Gielen, 1996b)(Gielen, 1996c). The *in vivo* toxicity profiles in mice and the antitumour activity in tumour bearing mice (Gielen, 1995) were screened for four di-n-butyltin carboxylates, di-n-butyltin bis(2,4-dihydroxybenzoate) (**1**), bis(2,5-dihydroxybenzoate) (**2**), bis(pentafluorophenylacetate) (**3**), and bis[di-n-butyl(pentafluorophenylacetato)tin] oxide (**4**) (Gielen, 1996d).
All compounds revealed similar *in vitro* chemosensitivities in two cell lines, C26-10 and C26-A, two murine undifferentiated colon carcinoma cell lines. With all compounds tested, not only cell growth was inhibited *in vitro*, but also cell kill was achieved. At their maximum tolerated dose (MTD), **1** and **4** were inactive *in vivo* against colon 26 tumours in Balb/C mice whereas compounds **2** and **3** showed slight *in vivo* antitumour activity. However, the cut-off level for the growth delay factor (GDF) (>1) was not reached. With the exception of **2** administered with a single dose and **3** with the 2 doses protocol, treatment with these compounds did not increase the life span of all mice. Repeated administration of compound **2** did not improve the antitumour activity compared to single dose administration.
An additional set of compounds, active *in vitro*, four di-n-butyltin and five triorganotin carboxylates, namely di-n-butyltin diterebate (**5**) (Gielen, 1997), bis(phenylacetate) (**6**), bis(deoxycholate) (**7**) (Willem, 1997a), bis(lithocholate) (**8**) (Willem, 1997a), tri-n-butyltin terebate (**9**) (Gielen, 1997), cinnamate (**10**) (Willem, 1997b), and triphenyltin terebate (**11**) (Gielen, 1997), were subjected to further investigations. *In vivo* toxicity and antitumour screening results are presented.

### Materials and Methods

The structures of the seven compounds screened are depicted in figure 1. Their synthesis and characterization were described earlier (Gielen, 1997)(Willem, 1997a) (Willem, 1997b) .

*Toxicity and in vivo screening.*
The experimental procedure was described earlier (Gielen, 1996d).
The test compounds were dissolved in DMSO to a concentration ranging from 50 to 100 mg/ml and diluted to 10 mg/ml in arachidis oil, which was also used for further dilutions. Occasionally the

DMSO had to be acidified or made alkaline to get a clear solution. The amount of DMSO could not be increased since mice do not tolerate more than 1%. Mice were injected intra-peritoneally.
The MTD was defined as the dose resulting in 10-15% weight loss.

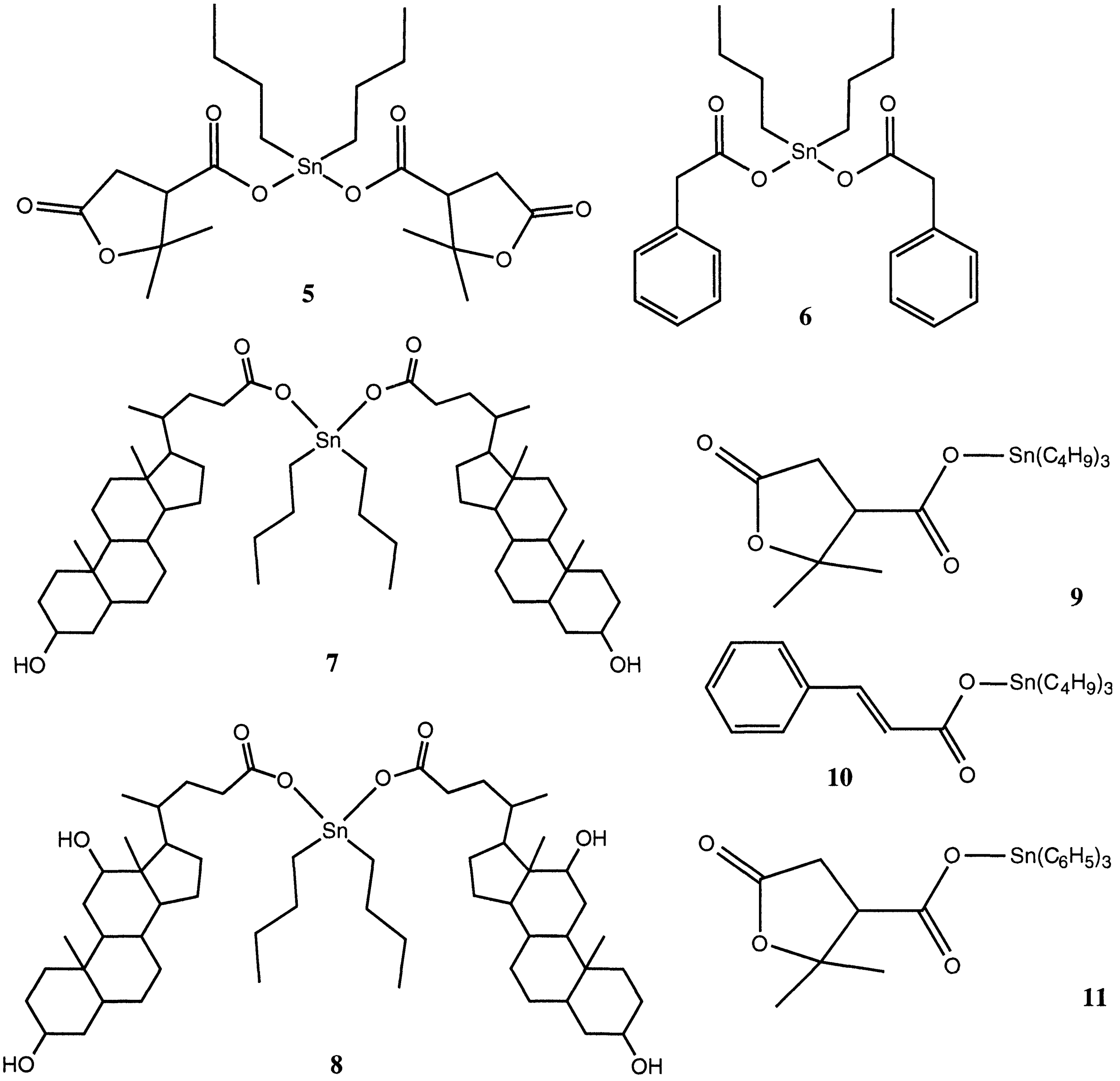

Figure 1: Structures of compounds **5** to **11**

*Antitumour activity.*

The murine colon tumour Colon 26 (Corbett, 1975) is maintained in our laboratory since 1983 (Peters, 1987, 1989; Van der Wilt, 1992) in Balb/c mice by s.c. transplantation in both flanks in the thoracic region of small fragments of 1-5 $mm^3$.

Several variants of the tumour have been characterized since its original characterization, all displaying different properties (summarized in Van Laar, 1996). The variant used in this work, originally described as colon 26, has been used throughout all experiments in our laboratory.

When tumours had reached a volume of 50-150 $mm^3$, treatment was started. Tumour size was determined by calliper measurement (length x width x height x 0,5) twice a week. The volume of the tumours was expressed relatively to that on the first day of treatment (day 0). Antitumour activity was evaluated by calculation of the T/C (ratio of the tumour size of the Treated mice to that of Control mice expressed in %) .

### Results and discussion

*Dose-finding studies of compounds **5** to **11** in MTD studies*

Initial dose-finding studies were performed with groups of 2 mice which were treated weekly for two weeks by i.p. injection (qd7x2). Results are shown in figures 2-8 for compounds **5** to **11**. For all tested compounds, a steep dose-toxicity relation was found. In addition, for the poorly soluble compounds **8** and **11**, the toxicity was unpredictable. For example, in a repeated experiment using the same dose (i.e. 20 mg/kg), a completely different toxicity compared to a former experiment was observed for these two compounds. This was probably due to the poor solubility of the compounds in DMSO resulting in a colloidal suspension in arachidis oil, resulting in non-reproducible injection of the compounds.

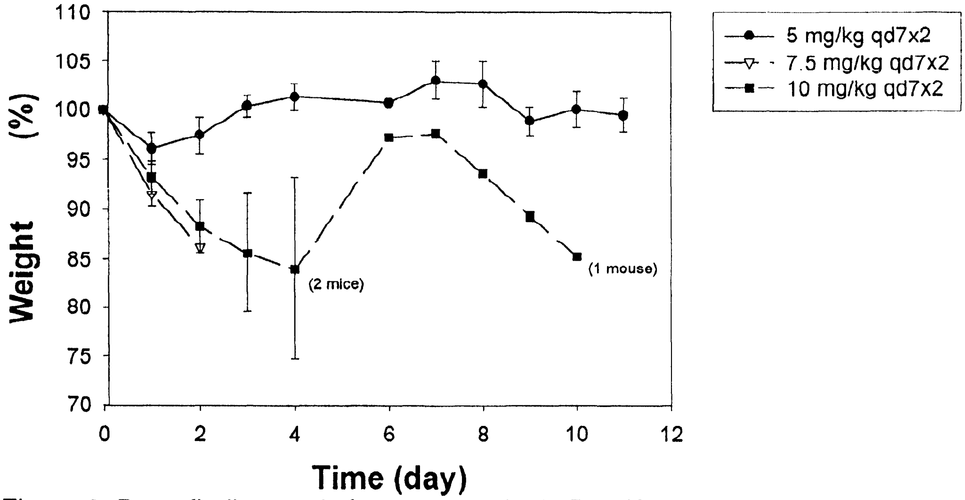

Figure 2: Dose-finding study for compound **5** in Balb/C mice

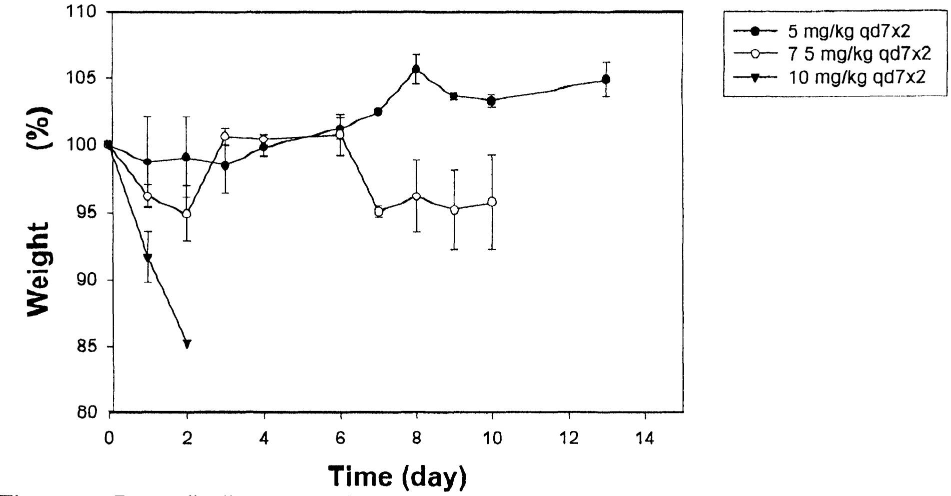

Figure 3: Dose-finding study for compound **6** in Balb/C mice

*Toxicity of compounds **5** to **11** in the antitumour activity studies*

Based on the results obtained in the dose-finding study, for each compound, a dose was chosen somewhat lower than the MTD dose, since experience with previous compounds demonstrated more toxicity in tumor bearing mice. The expected toxicity should be less than 10%.

However, the toxicity in tumor (colon 26A) bearing Balb/C mice was unexpectedly high (figures 9 -11). For the compounds **6**, **7**, **8**, **10** and **11**, severe toxicities were observed already after one injection. Therefore, administration of a second injection of these compounds was not acceptable. However, for compound **10**, 3/8 mice could be treated twice. The compounds **5** and **9** were administered according to the schedule qd7x2. For **9**, this schedule was too toxic (3/5 toxic deaths within 1 week).

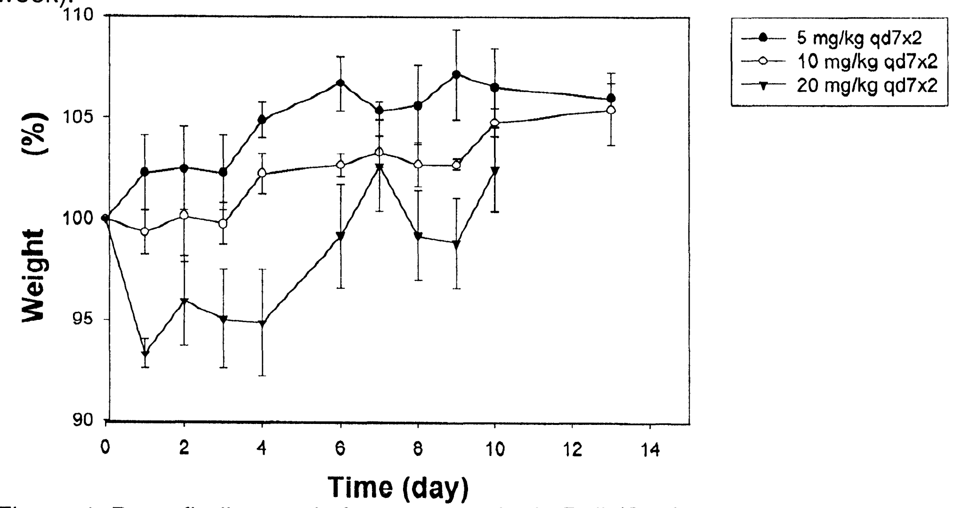

Figure 4: Dose-finding study for compound **7** in Balb/C mice

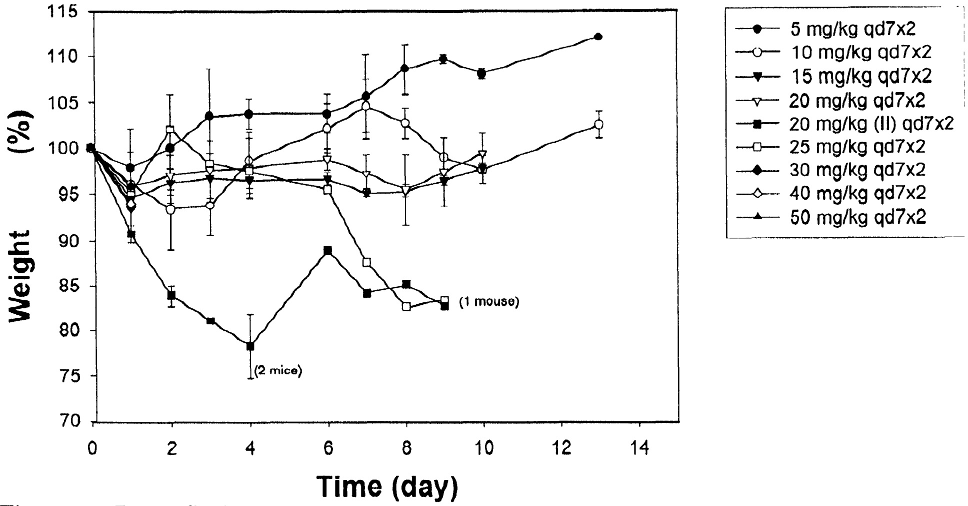

Figure 5: Dose-finding study for compound **8** in Balb/C mice

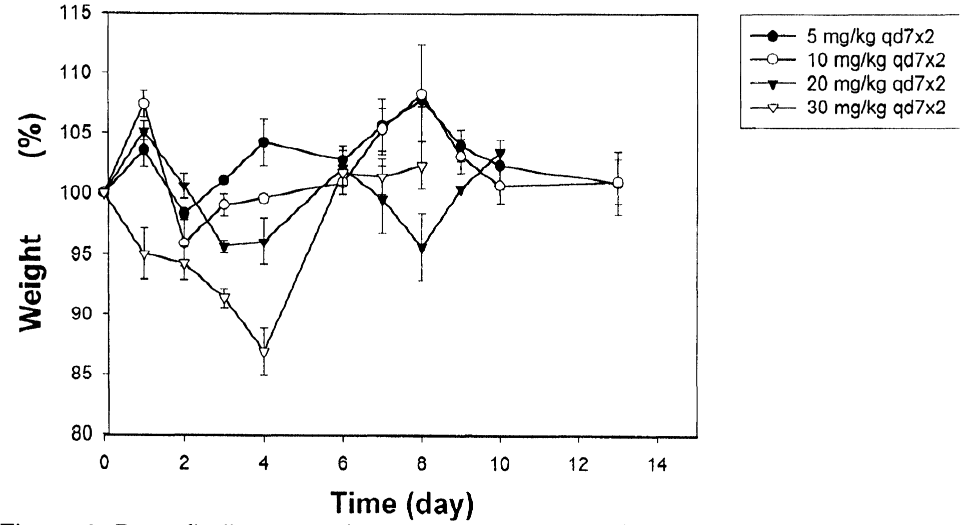

Figure 6: Dose-finding study for compound **9** in Balb/C mice

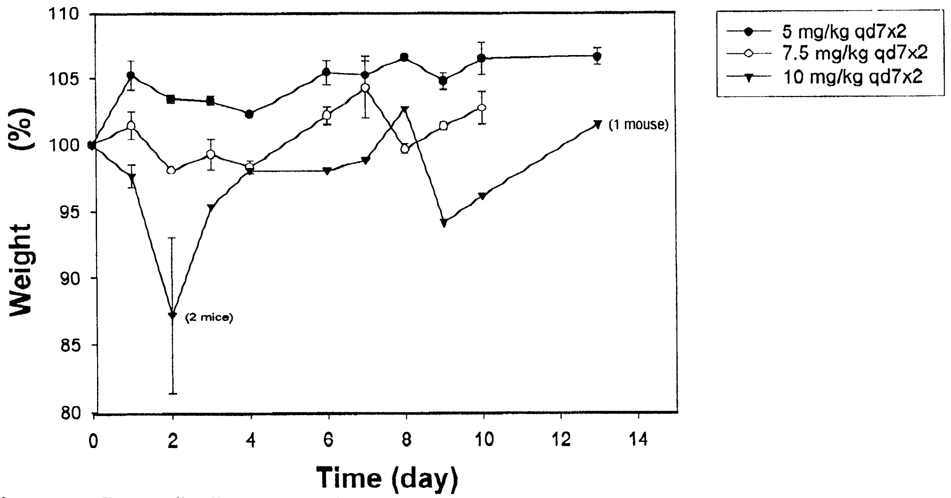

Figure 7: Dose-finding study for compound **10** in Balb/C mice

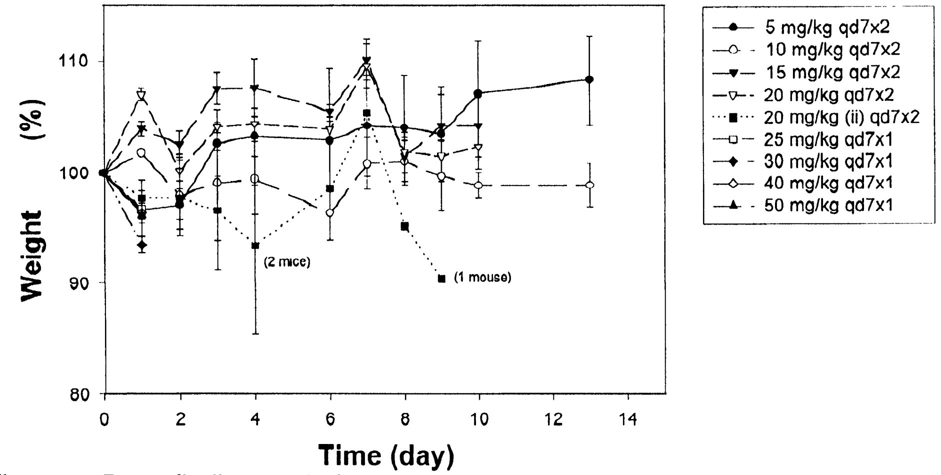

Figure 8: Dose-finding study for compound **11** in Balb/C mice

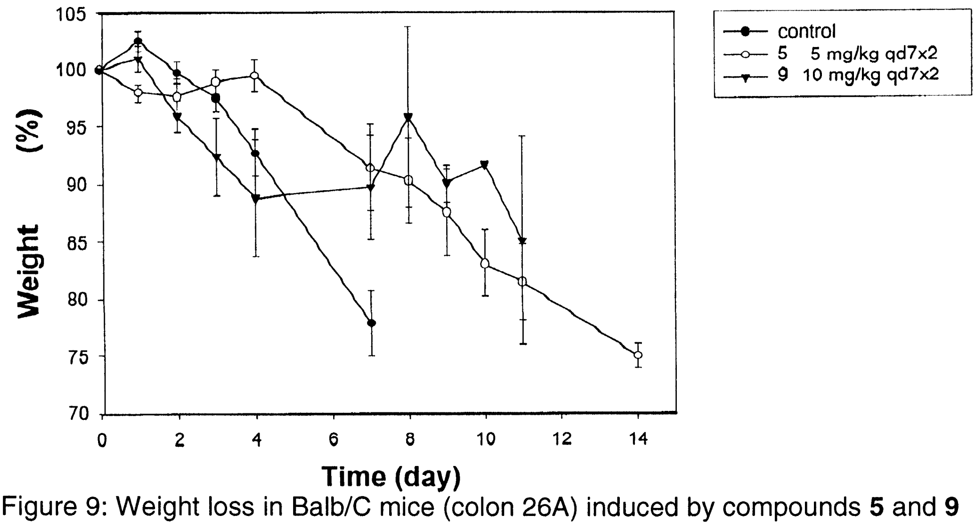

Figure 9: Weight loss in Balb/C mice (colon 26A) induced by compounds **5** and **9**

*In vivo antitumour activity*

Although the compounds were too toxic to administer according to the schedule used in the dose-finding study, conclusions can be drawn based on a single dose treatment of the colon26A bearing mice. The assumption was made that when even at this high dose no antitumour activity is present, it would be unlikely that lower doses at a schedule of qd7x2 would be able to produce an antitumour effect.

At their MTD, no antitumour effect was observed for the compounds **5**, **7**, **9**, **10** and **11**, i.e. the T/C ~1 (see fig. 12 to 14).

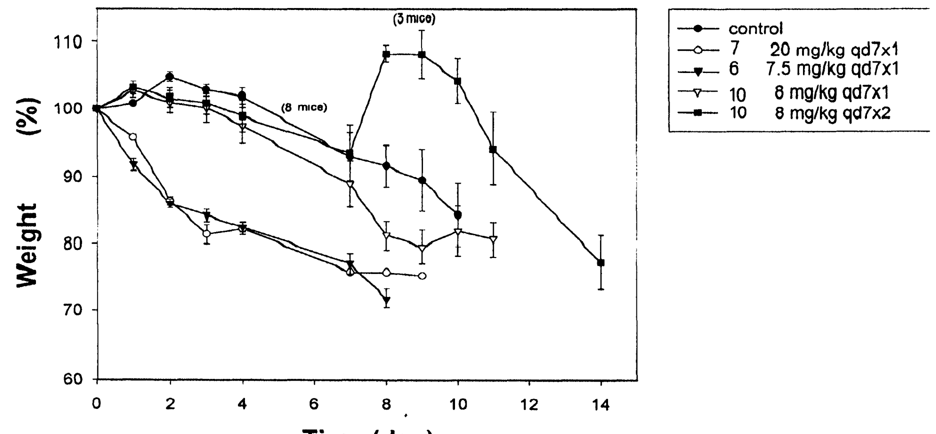

Figure 10: Weight loss in Balb/C mice (colon 26A) induced by compounds **6**, **7** and **10**

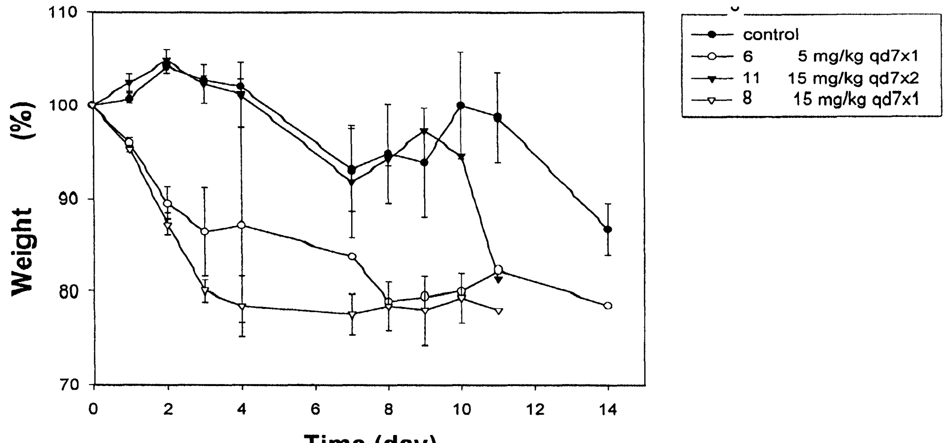

Figure 11: Weight loss in Balb/C mice (colon 26A) induced by compounds **6**, **8** and **11**

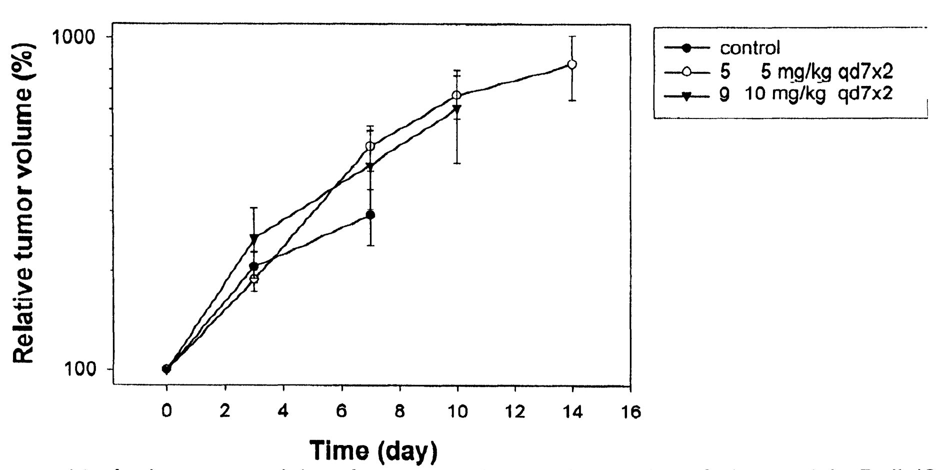

Figure 12: Antitumour activity of compounds **5** and **9** against Colon 26A in Balb/C mice

The compounds **6** and **8** (see fig. 13 and 14) showed clear antitumour activity after single dose administration (for **6** and **8**, T/C equals respectively 0.51 and 0.42). The dose for both compounds was too toxic, however. Because of the promising results obtained with compound **6** at a single dose of 7.5 mg/kg (fig 13), the dosis was decreased to 5 mg/kg in a second experiment (fig. 14) in order to reduce the toxicity. The reduced dose also showed a antitumour effect which was less (T/C ~ 0.81 ) but showed a comparable severe toxicity.

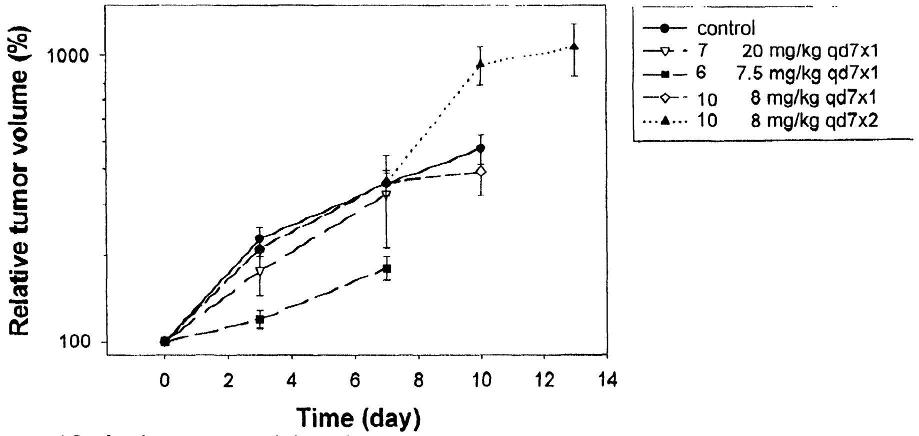

Figure 13: Antitumour activity of compounds **6**, **7** and **10** against Colon 26A in Balb/C mice

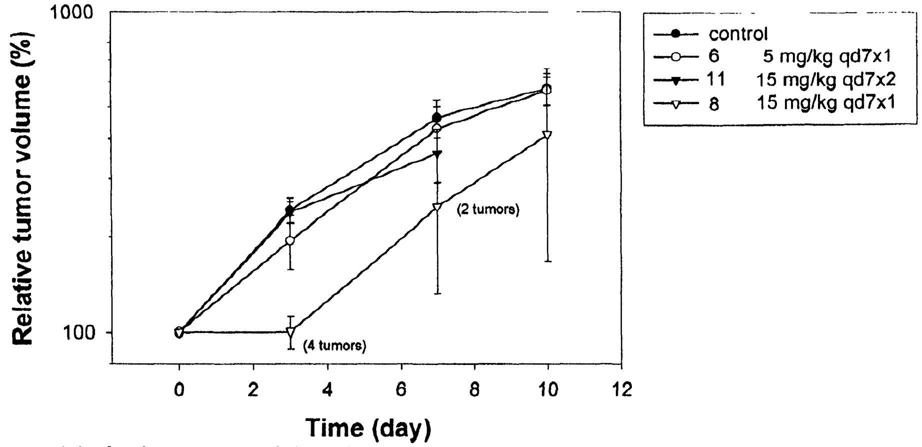

Figure 14: Antitumour activity of compounds **6**, **8** and **11** against Colon 26A in Balb/C mice

### Conclusions

The compounds **6** and **8** might be of interest for further antitumour activity studies. However, their severe toxicity makes the results difficult to interpret. An adapted formulation for administration of the compounds will be necessary for further evaluation of possible antitumour effects.

### Acknowledgements

This research was supported by the Belgian "Nationaal Fonds voor Wetenschappelijk Onderzoek" (N.F.W.O. grant number S2/5 CD F198, M.G.), the Belgian "Fonds voor Kollektief Fundamenteel Onderzoek" (F.K.F.O. grant number 2.0094.94, R.W.), the Belgian "Nationale Loterij" (grant number 9.0006.93, R.W.) and the "Fund for Scientific Research Flanders" (Belgium, Grant G0192.98, R.W.).

### References

Bouâlam M, Gielen M, Meriem A, de Vos D, Willem R, Pharmachemie B.V., *Eur. Pat.* Publ. 472 783, Appl. 90/202,316.7, 21/09/90, Anti-tumor compositions and compounds, *Chem.Abstr.* (1992) **116**: 235 873q

Bouâlam M, Gielen M, El Khloufi A, de Vos, D., Willem, R., Pharmachemie B.V., *Eur. Pat.* Publ. 538 517, Appl. 91/202,746.-, 22.10.91, Novel organo-tin compounds having anti-tumour activity and anti-tumour compositions, *Chem. Abstr.* (1993) **119**: 117 548b

Corbett TH, Griswold DP, Roberts BJ, Peckham JC, Schabel FM. Evaluation of single agents and combinations of chemotherapeutic agents in mouse colon carcinomas. *Cancer* (1975) **40**: 2660-2680

Gielen M, Pan H, Willem R, de Vos D, Pharmachemie B.V., *Eur. Pat.* Publ. 484 596, Appl. 90/202,936.2, 06/11/90, Antitumour compositions and compounds, *Chem. Abstr.* (1992)**117**, 111 807x

Gielen M, Willem R, Bouhdid A, de Vos D, Kuiper CM, Veerman G, Peters GJ, *In vitro* antiproliferative effects, toxicity profiles *in vivo* in mice and antitumour activity in tumour-bearing mice of five organotin compounds, *In vivo* (1995) **9**: 59-64

Gielen M, Willem R, Bouhdid A, de Vos D, Pharmachemie B.V., *Eur. Pat.* Publ. 677 528 A1, Appl. 94/201012.5, 12/04/94, Dibutyltin bis(hydroxybenzoates) and compositions containing these compounds, *Chem. Abstr.* (1996a) **124**: 87 376c

Gielen M, Willem R, Bouhdid A, de Vos D, Pharmachemie B.V., *Eur. Pat.* Publ. 700 921, Appl. 94/202,612.1, 09/09/94, Aromatic fluorine-containing organotin compounds and anti-tumour compositions, *Chem. Abstr.* (1996b) **124**: 343 674z

Gielen M, Tin-based antitumour drugs, *Coord. Chem. Rev.* (1996c) **151**: 41 - 51

Gielen M, Willem R, Bouhdid A, de Vos D, Kuiper CM, Veerman G, Peters GJ, *In vitro* antiproliferative effects, toxicity profiles *in vivo* in mice and antitumour activity in tumour-bearing mice of four diorganotin compounds, *Oncol. Rep.* (1996d) **3**: 583 - 587

Gielen, M, Ma H, Bouhdid A, Dalil H, Biesemans M, Willem R, Di-n-butyl-, tri-n-butyl- and triphenyltin dl-terebates: synthesis, characterization and *in vitro* antitumour activity, *Metal-Based Drugs* (1997) **4**: 193 - 197

Peters GJ, Van Dijk J, Nadal J, Van Groeningen CJ, Lankelma J, Pinedo HM. Diurnal variation in the therapeutic efficacy of 5-fluorouracil against murine colon cancer. *In Vivo* (1987) **1**: 113-118

Peters GJ, Braakhuis BJM, De Bruijn EA, Laurensse EJ, Van Walsum M, Pinedo HM. Enhanced therapeutic efficacy of 5'-deoxy-5-fluorouridine in 5-fluorouracil resistant head and neck tumours in relation to 5-fluorouracil metabolizing enzymes, *Brit. J. Cancer* (1989) **59**: 327-334

Van der Wilt CL, Smid K, Pinedo HM, Peters GJ. Elevation of thymidylate synthase following 5-fluorouracil treatment is prevented by addition of leucovorin in murine colon tumors, *Cancer Res.* (1992a) **52**: 4922-4928

Van Laar JAM, Rustum YM, Van der Wilt C, Smid K, Kuiper CM, Pinedo HM, Peters GJ. Tumor size and origin determine the antitumor activity of cisplatin or 5-fluorouracil and its modulation by leucovorin in murine colon carcinomas, *Cancer Chemother. Pharmacol.* (1996) **39**: 79-89

Willem R, Dalil H, Broekaert P, Biesemans M, Ghys L, Nooter K, de Vos D, Ribot F, Gielen M, Di-n-butyl-, tri-n-butyl- and triphenyltin steroidcarboxylates: synthesis, NMR characterization and *in vitro* antitumour activity, *Main Group Met. Chem.* (1997a) **20**: 535 - 542

Willem R, Bouhdid A, Mahieu B, Ghys L, Biesemans M, Tiekink ERT, de Vos D, Gielen M, Synthesis, characterization and *in vitro* antitumour activity of triphenyl- and tri-n-butyltin benzoates, phenylacetates and cinnamates, *J. Organomet. Chem.* (1997b) **531**:151 - 158

**Received: January 21, 1998 - Accepted: February 12, 1998 - Received in revised camera-ready format: February 12, 1998**

